# Supplementary material for: Biocompatible Carbon Dots/Polyurethane Composites as Potential Agents for Combating Bacterial Biofilms: N-Doped Carbon Quantum Dots/Polyurethane and Gamma Ray-Modified Graphene Quantum Dots/Polyurethane Composites
Source: Pharmaceutics. 2024 Dec 6;16(12):1565. doi: 10.3390/pharmaceutics16121565 (PMC11676340; doi:10.3390/pharmaceutics16121565)
Supplement: Supplementary file 1 [file pharmaceutics-16-01565-s001.zip › pharmaceutics-3252994-supplementary.pdf]

Supporting information

Biocompatible carbon dots/polyurethane composites as potential agents combating bacteria biofilms: N-doped carbon quantum dots/polyurethane and gamma rays modified graphene quantum dots/polyurethane composites case

Z. Marković[1*], S. Dorontić[1], S. Jovanović[1], J. Kovač[2], D. Milivojević[1], D. Marinković[1], M. Mojsin[3], B. Todorović Marković[1*]

[1]Vinča Institute of Nuclear Sciences, National Institute of the Republic of Serbia, University of Belgrade, Mike Petrovića Alasa 12-14, 11001 Belgrade, Serbia

[2]Jozef Stefan Institute, Department of Surface Engineering, Jamova 39, SI-1000 Ljubljana, Slovenia

[3]Institute of Molecular Genetics and Genetic Engineering, University of Belgrade, Vojvode Stepe 444a, 11042 Belgrade, Serbia

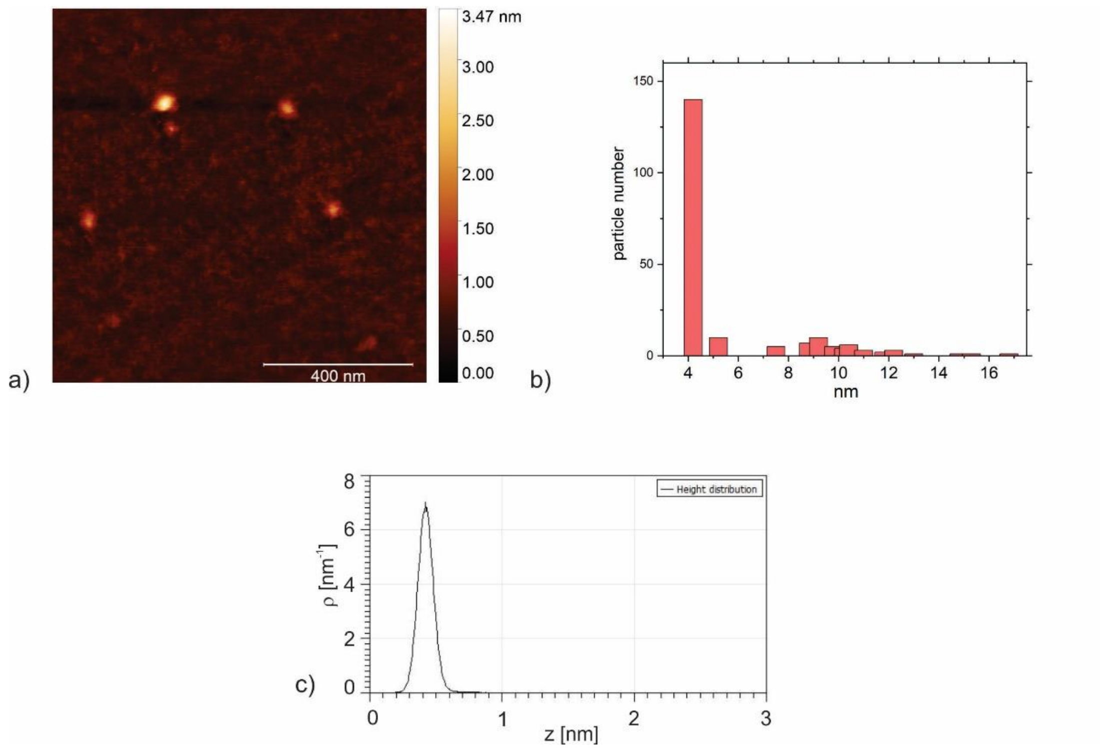

Figure S1. a) Top view AFM image of CAUR-CQDs nanoparticles, b) particle size distribution of CAUR-CQDs nanoparticles, c) height profile of CAUR-CQDs nanoparticles.

* Correspondence: zoranmarkovic@vin.bg.ac.rs (Z. M. M.) biljatod@vin.bg.ac.rs (B.M.T.M.); Tel.: +381 113408582

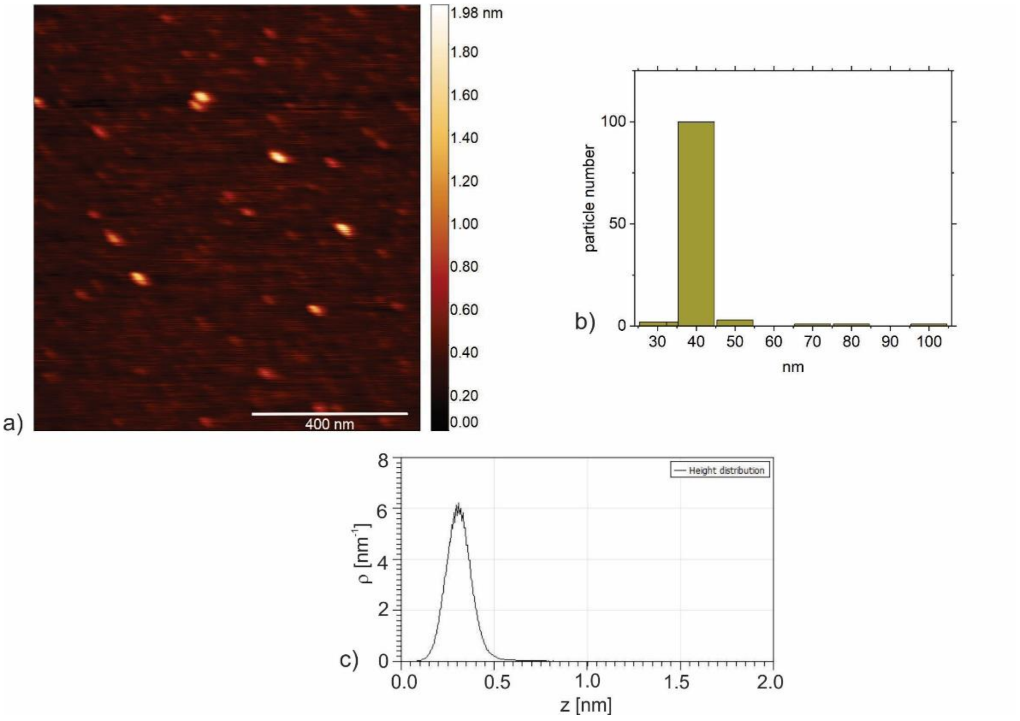

Figure S2. a) Top view AFM image of GQD50 nanoparticles, b) particle size distribution of GQD50 nanoparticles, c) height profile of GQD50 nanoparticles.

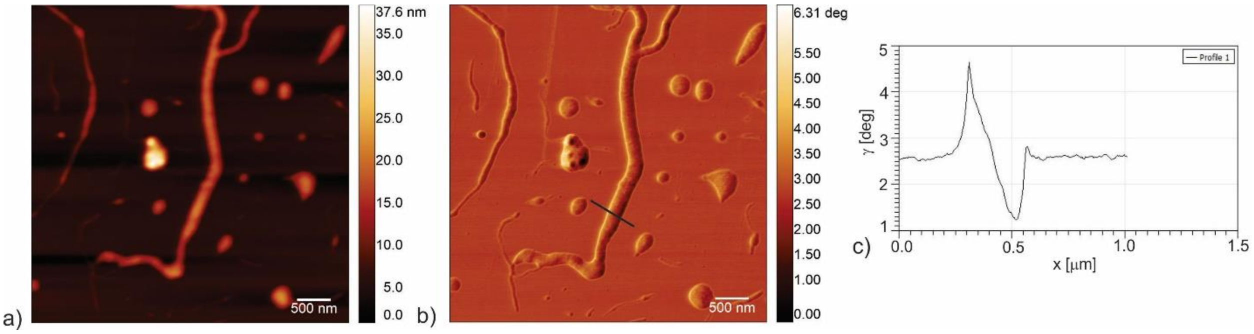

Figure S3. EFM images of SWCNT (6,5): a) height retrace mode, b) nap retrace mode, c) charge distribution profile of SWCNT (6,5) (black line on Figure S3b).

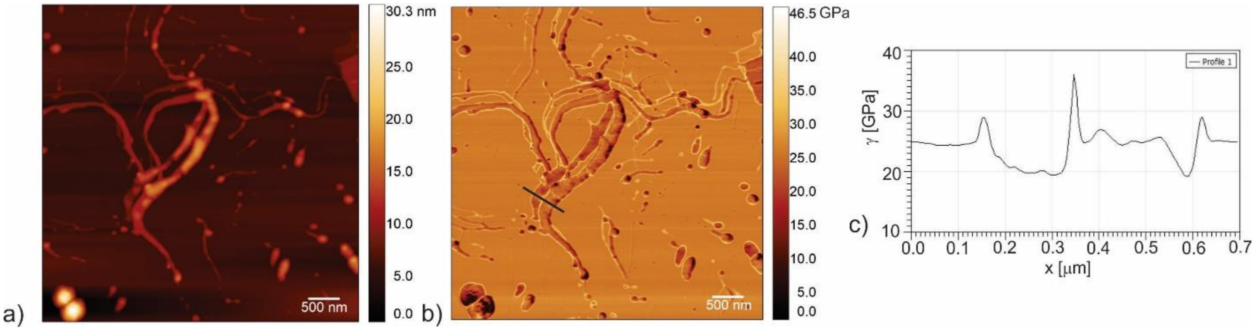

Figure S4. AMFM images of SWCNT (6,5,): a) height retrace mode, b) Young's retrace, c) profile of Young's modulus of elasticity of SWCNT.

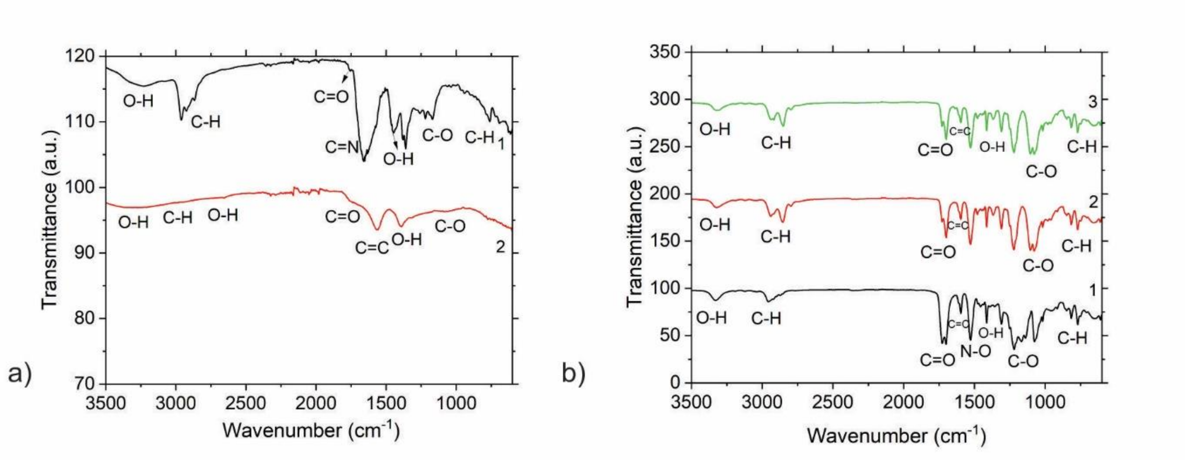

Figure S5. a) FTIR spectra of CAUR-CQDs (black curve 1) and GQD50 (red curve 2) nanoparticles, b) FTIR spectra of neat PU (black curve), b) CAUR-CQDs/PU (red curve) and c) GQD50/PU (green curve) composite films.

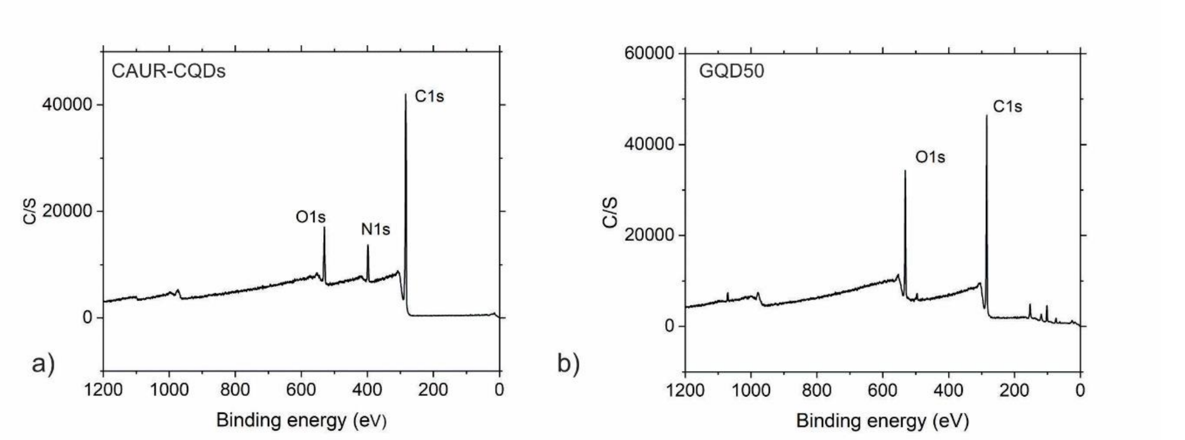

Figure S6. XPS survey spectra of a) CAUR-CQDs and b) GQD50 nanoparticles.

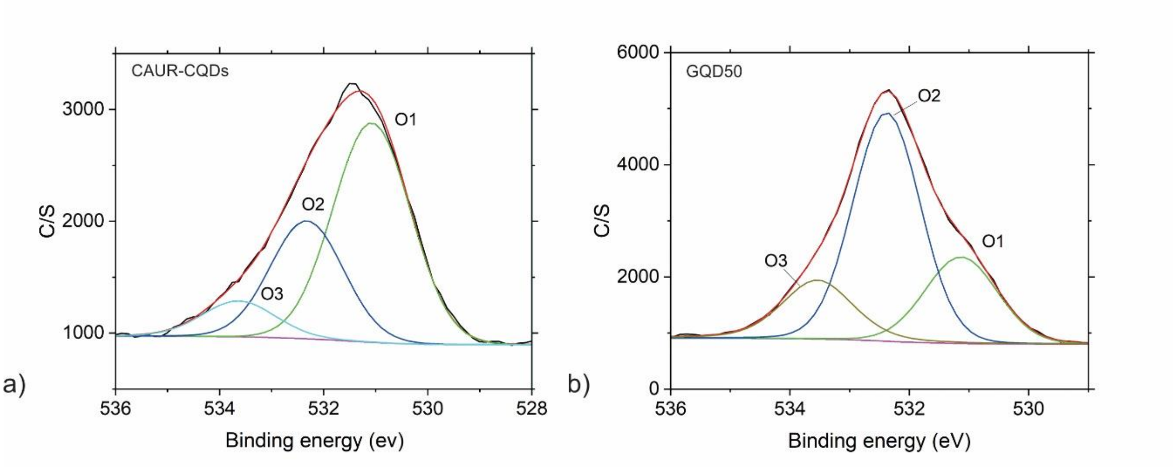

Figure S7. XPS deconvoluted O1s spectra of a) CAUR-CQDs and b) GQD50 nanoparticles.

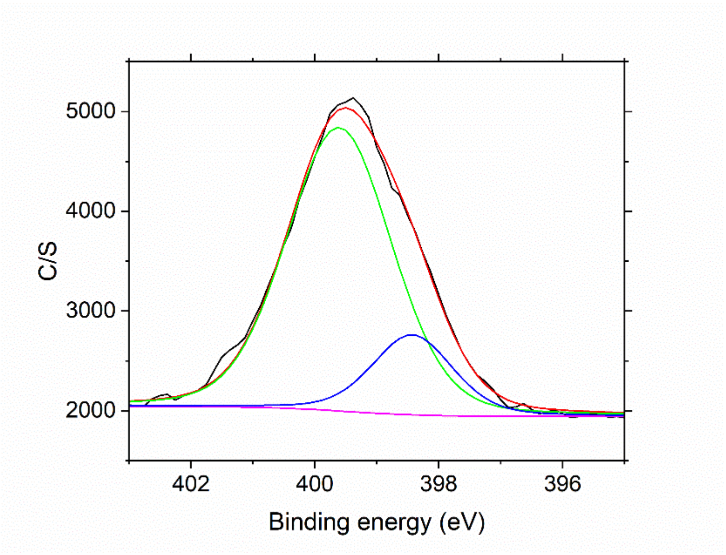

Figure S8. XPS deconvoluted N1s spectra of CAUR-CQDs nanoparticles.

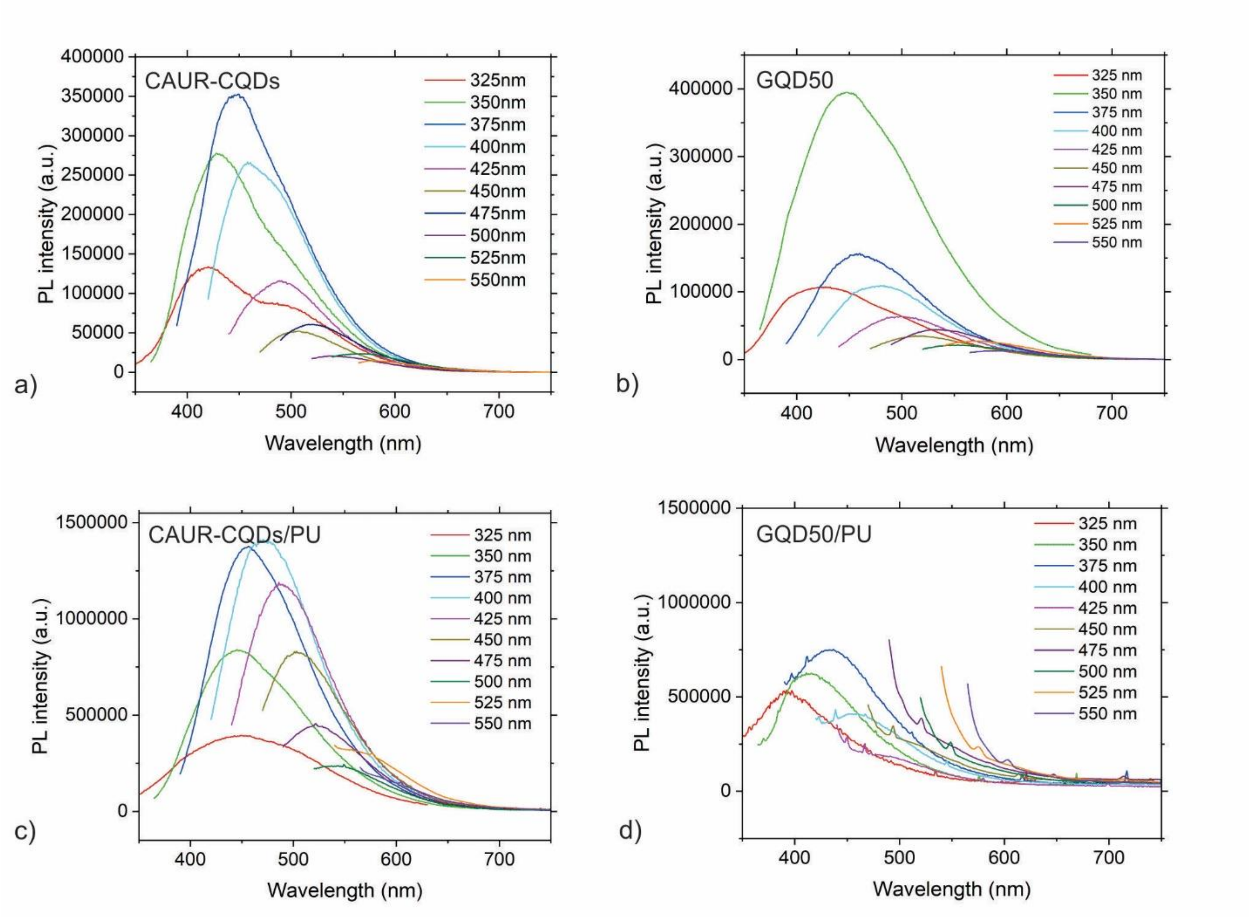

Figure S9. PL spectra of a) CAUR-CQDs, b) GQD50 nanoparticles; PL spectra of c) CAUR-CQDs/PU and d) GQD50 composite films.

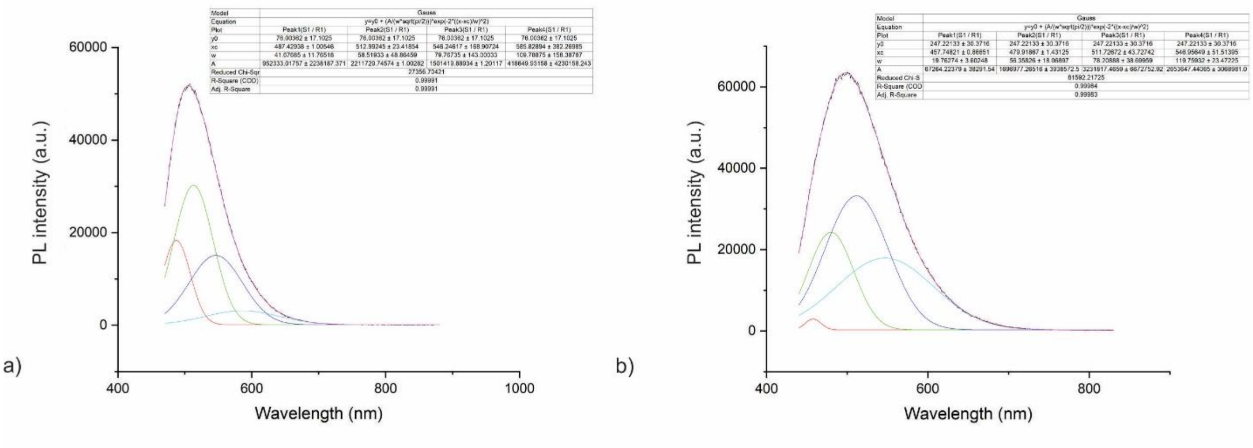

Figure S10. Fitted PL spectra of a) CAUR-CQDs and b) GQD50 nanoparticles. The excitation wavelengths for each sample were 450 nm.

Table S1. Antibacterial activity of neat PU films.

PU	Exposed to blue light for 1 h (irradiated)	Not exposed to blue right (non-irradiated)
	^{a}N (cell/cm^2)	^{a}N (cell/cm^2)
S. aureus	4 x10^8	4.5x10^8
MRSA	2 x10^8	3.2x10^8
E. faecalis	4.5 x10^6	2.7x10^7
P. aeruginosa	3.8 x10^{10}	3.4x10^{10}
K. pneumonie	2.6 x10^6	1.8x10^6
L. monocytogenes	1.7 x10^6	1.3x10^6
E. coli	3.2 x10^{10}	3.0x10^{10}
A.baumanii	3.9 x10^8	2.9x10^8

^{a}N = the number of viable bacteria recovered per cm^2 per test sample.

Table S2**.** Antibacterial activity of CAUR-CQDs/PU composite films.

CAUR-CQDs/PU	Exposed to blue light for 1 h(irradiated)		Not exposed to blue right (non-irradiated)
	^{a}N (cell/cm^2)	^{b}R$_{\text{compared to non-irradiated PU}}$	^{a}N (cell/cm^2)
S. aureus	5	5.2	5x10^8
MRSA	5	4.3	4.4 x10^8
E. faecalis	5	4.7	4 x10^8
P. aeruginosa	32x 10^9	0.06	28x10^9
K. pneumonie	5	5.3	2.5 x10^6
L. monocytogenes	3.8x10^6	0.06	12x10^6
E. coli	73x10^9	0.02	78x10^9
A.baumanii	5	4.9	5.3 x10^8

^{a}N = the number of viable bacteria recovered per cm^2 per test sample. ^{b}R = the antibacterial activity.

Table S3. Antibacterial activity of GQD50/PU composite films.

GQD50/PU	Exposed to blue light for 1 h (irradiated)		Not exposed to blue right (non-irradiated)
	[a]N (cell/cm^2)	[b]$R_{\text{compared to non-irradiated PU}}$	[a]N (cell/cm^2)
S. aureus	26x10^9	0.13	36x10^9
MRSA	32 x10^7	0.5	1.5x10^5
E. faecalis	3.5x10^4	1.12	4.6x10^5
P. aeruginosa	27 x10^{10}	0.05	31x10^9
K. pneumonie	2.8x10^6	1.5	5.5x10^5
L. monocytogenes	1.9x10^6	1.04	18x10^6
E. coli	95 x10^9	0.02	50x10^9
A.baumanii	8.8x10^6	0.96	7.8x10^6

[a]N = the number of viable bacteria recovered per cm^2 per test sample. [b]R = the antibacterial activity.
